# singleCellBase: a high-quality manually curated database of cell markers for single cell annotation across multiple species

**DOI:** 10.1186/s40364-023-00523-3

**Published:** 2023-09-20

**Authors:** Fan-Lin Meng, Xiao-Ling Huang, Wen-Yan Qin, Kun-Bang Liu, Yan Wang, Ming Li, Yong-Hong Ren, Yan-Ze Li, Yi-Min Sun

**Affiliations:** 1 Marketing and Management Department, CapitalBio Technology, Beijing, 100176 China; 2 National Engineering Research Center for Beijing Biochip Technology, Beijing, 102206 China; 3 Neonatal Screening Center, Dongguan Maternal and Child Healthcare Hospital, Dongguan, 532002 China

**Keywords:** Single-cell RNA-seq, Cell annotation, Database

## Abstract

Annotating cells in the analysis of single-cell RNA-seq (scRNA-seq) data is one of the most challenging tasks that researchers are actively addressing. Manual cell annotation is generally considered the gold standard method, although it is labor intensive and independent of prior knowledge. At present, the relationship between high-quality, known marker genes and cell types is very limited, especially for a variety of species other than humans and mice. The singleCellBase is a manually curated resource of high-quality cell types and gene markers associations across multiple species. In details, it offers 9,158 entries spanning a total of 1,221 cell types and linking with 8,740 genes (cell markers), covering 464 diseases/status, and 165 types of tissues across 31 species. The singleCellBase provides a user-friendly interface to the scientific community to browse, search, download and submit records of marker genes and cell types. The resource providing ineluctable prior knowledge required by manual cell annotation, which is valuable to interpret scRNA-seq data and elucidate what cell type or cell state that a cell population represents.

**Supplementary Information:**

The online version contains supplementary material available at 10.1186/s40364-023-00523-3.

## Main text

Dear Editor (s),

The annotation of cells in single-cell RNA-seq (scRNA-seq) data analysis poses a significant challenge for researchers [1–4]. While manual cell annotation is widely regarded as the gold standard, its labor-intensive nature and lack of reliance on prior knowledge present limitations. Existing databases and tools, such as PanglaoDB [5], CellMarker v2.0 [6], and SingleR [7], have made valuable contributions. However, these resources primarily focus on a limited range of species, with an emphasis on humans and mice, leaving a gap in knowledge. To bridge this gap, we developed the singleCellBase, a meticulously curated resource that provides high-quality associations between cell types and gene markers across multiple species. It is freely available at http://cloud.capitalbiotech.com/SingleCellBase/.

We meticulously collected targeted and representative associations of cell types and gene markers by utilizing the curated publications available on the 10x Genomics website (https://www.10xgenomics.com/resources/publications) as our primary source. This approach was chosen to take advantage of the high quality and relevance offered by the 10x Genomics platform. Our data collection process involved several steps. Firstly, we performed a preliminary review of all the literature, reading abstracts to remove irrelevant articles. Secondly, we manually surveyed and read through each full text and relevant Supplement Tables. During this process, we extracted the associations of cell types and gene markers, along with the descriptions of the supporting evidence. Thirdly, the curated associations were carefully double-checked to ensure their accuracy. Additionally, we put significant effort into normalizing and unifying the names of all cell types, tissue or organ names, and disease names separately. Currently, the singleCellBase contains 9,158 entries, encompassing 1,221 cell types linked to 8,740 gene markers, and covering 464 diseases/states and 165 tissue types across 31 species. Each entry provides detailed information on cell types, gene markers, tissue or organ names, disease or state names, confidence level of the associations, PubMed ID, journal, related datasets, evidence description and other information.

**Fig. 1:**
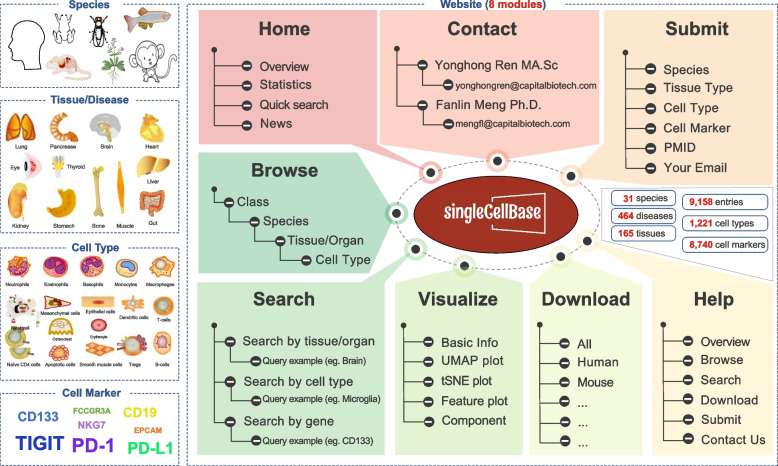
The schematic workflow of the singleCellBase database

**Fig. 2:**
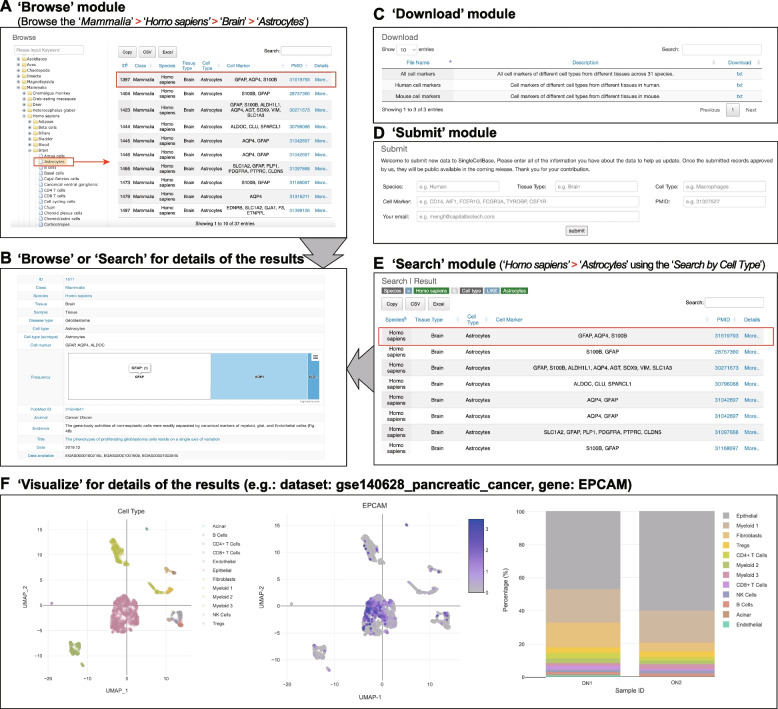
The user interfaces of each functional module in the singleCellBase database. **A** An interface of the ‘*Browse’* module. **B** An interface of the detailed results of “*Browse*” or “*Search*” module. **C** An interface of the “*Download*” module. **D** An interface of the “*Submit*” module. **E** An interface of the “*Search*” module. **F** An interface of the “*Visualize*” module for details of the results

The singleCellBase database offers user-friendly web interfaces that facilitates easy browsing, searching, visualizing, downloading, submitting the associations of cell type and gene marker. Figure 1 illustrates the schematic workflow of the singleCellBase database. In the “*Browse*” page, users can navigate through species using a taxonomic classification system, explore tissues or organs, and explore cell types in a hierarchical structure (Fig. 2A and Table S1). To improve user efficiency, we have implemented fuzzy search tools that provide a concise browsing result page with matched entries. Additionally, users can access detailed information for each entry by clicking the “*More*” button (Fig. 2B). In the “*Download*” page (Fig. 2C), users can download files in a text format, facilitating easy access to the data. Furthermore, users have the added convenience of accessing result data effortlessly via the result interface found in both the “Browse” and “Search” modules. This feature not only facilitates personalized querying but also empowers users to download result tables after performing a search. In the “*Submit*” page (Fig. 2D), users have the ability to contribute their own associations of cell types and gene markers to the singleCellBase database, promoting collaboration and expanding the available knowledge.

In the “*Search*” page, it offers users three different ways to search through all entries by tissue type, cell type, or gene markers (Fig. 2E). For example, within the search interface, users can retrieve associations between cell types and gene markers in a specific species. In the “*Search by Cell Marker*” page, users can explore cell types associated with the *CD8* gene marker in humans. By selecting the “*Homo sapiens*” species and entering “*CD8A*” as the gene marker keyword, users can investigate the cell types linked to *CD8*. Consistent with previous studies, *CD8* is predominantly expressed in *CD8* T cells in humans. To ensure the reliability of the associations, we calculate the frequency of cell type and gene marker associations in scientific literature and present the results graphically. This allows users to easily identify high-confidence associations between cell types and gene markers. The search module in the singleCellBase database empowers users to efficiently explore the connections between cell types and gene markers, providing valuable insights and facilitating further research in this field.

To facilitate a deeper exploration of gene of interest expression patterns in scRNA-seq data, we have developed the “*Visualize*” module (Fig. 2F). When utilizing the “*Visualize*” module, users are initially prompted to select a scRNA-seq dataset and inputting gene of interest that provides expression patterns at the single-cell level. As result, there are several graphical results, such as UMAP/t-SNE, Feature plot based on UMAP/t-SNE, barplot to show cell component. In a previous study conducted by Zheng et al., flow cytometry was utilized to successfully sort epithelial cells (CD45-EPCAM +) in pancreatic cancer [8]. By applying the “*Visualize*” module in our database, we have further validated the specific expression of EPCAM in epithelial cells. This module empowers users to effectively explore and visualize gene expression patterns in scRNA-seq data. By leveraging the provided visualization tools and datasets, researchers can gain valuable insights into the spatial organization and cellular composition within specific biological contexts.

In summary, the singleCellBase database is a valuable resource that provides gene markers for manual annotation across multiple species, encompassing the Animalia, Protista, and Plantae kingdoms. This resource offers essential prior knowledge necessary for the manual annotation of cells, aiding in the interpretation of scRNA-seq data and providing insights into the cell types or states represented by a cell population. Notably, the singleCellBase stands out from other databases in several ways: (1) Wide species coverage: The database includes an extensive range of species, totaling 31, spanning both plants and animals. This diverse collection encompasses humans, mice, fish, chickens, monkeys, pigs, *Arabidopsis*, and more. (2) Accurate and abundant entries: With approximately 10,000 entries, the database ensures accuracy and credibility. These records are sourced from reliable literature resources such as the 10X Genomics websites and undergo rigorous double-checking during manual curation. (3) User-friendly interface: The singleCellBase offers an intuitive interface that facilitates browsing, searching, and visualization of cell types and markers through various functional components, namely Browse, Search, Visualize, Download, and Submit. Researchers can easily navigate and access the desired information. (4) Convenience: By eliminating the barriers associated with manual cell annotations in scRNA-seq analysis, our database provides a convenient solution for the research community, streamlining their workflows. (5) Unification: To address the specificity of cell types across different datasets, we have unified identical cell types under a single unified name during the collection of cell type and gene marker associations, ensuring consistency and ease of use. Overall, the singleCellBase database serves as a valuable resource for exploring markers associated with diverse cell types across multiple species. It empowers researchers in their investigations and contributes to the advancement of biological knowledge.

### Supplementary Information

**Additional file 1: Table S1.** The information pertains to the taxonomic classification of species.

## Data Availability

All data for this article can be found online at http://cloud.capitalbiotech.com/SingleCellBase/.

## Abbreviations

scRNA-seq: Single-cell RNA-seq
t-SNE: T-distributed stochastic neighbor embedding
UMAP: Uniform manifold approximation and projection
