# Supplementary material for: singleCellBase: a high-quality manually curated database of cell markers for single cell annotation across multiple species
Source: Biomark Res. 2023 Sep 20;11:83. doi: 10.1186/s40364-023-00523-3 (PMC10510128; doi:10.1186/s40364-023-00523-3)
Supplement: Supplementary file 1 — Additional file 1: Table S1. The information pertains to the taxonomic classification of species. [file 40364_2023_523_MOESM1_ESM.docx]

**Supplementary Files**

**singleCellBase: a high-quality manually curated database of cell markers for single cell annotation across multiple species**

Fan-Lin Meng^1, 2†^, Xiao-Ling Huang^3†^, Wen-Yan Qin^1, 2^, Kun-Bang Liu^1^, Yan Wang^1^, Ming Li^1^, Yong-Hong Ren^1*^, Yan-Ze Li^1*^, Yi-Min Sun^1, 2*^

**Database implementation**

In our singleCellBase, all data was organized using MySQL 14.14 based on relational schema, which will be supported to future singleCellBase updates. The website code was written based on Java Server Pages using the Java Servlet framework. The website is deployed on the Tomcat 6.0.44 web server and runs on a CentOS 5.5 Linux system. The JQuery was used to generate, render and manipulate data visualization. The singleCellBase website has been fully tested in Google Chrome and Safari browsers.

**Table S1.** The information pertains to the taxonomic classification of species.

| ID | List in singleCellBase | **Species** | **Genus** | **Family** | **Order** | **Cass** | **Phylum** | **Kingdom** |
| --- | --- | --- | --- | --- | --- | --- | --- | --- |
| 1 | Arabidopsis | Arabidopsis thaliana | Arabidopsis | Brassicaceae | Brassicales | Magnoliopsida | Angiospermae | Plantae |
| 2 | Axolotl |  |  |  |  |  |  |  |
| 3 | Caenorhabditis elegans | Caenorhabditis elegans | Caenorhabditis | Rhabditoidea | Rhabditidia | Rhabditia/Secernentea | Nematoda | Animalia |
| 4 | Chicken | Chicken | Gallus | Phasianidae | Galliformes | Aves | Chordata | Animalia |
| 5 | Ciona intestinalis | Intestinalis | Ciona | Cionidae | Phlebobranchia | Ascidiacea | Phylum Chordata | Animalia |
| 6 | Cnomolgus monkey | Cnomolgus monkey | Macaca | Cercopithecidae | Primates | Mammalia | Chordata | Animalia |
| 7 | Deer | Deer | Cervus | Cervidae | Artiodactyla | Mammalia | Chordata | Animalia |
| 8 | Drosophila | Drosophila hilidae | Drosophila | Drosophilidae | Diptera | Insecta | Arthropoda | Animalia |
| 9 | Earthworm | Lumbricus terrestris | Lumbricus | Lumbricidae | Opisthopora | Chaetopoda | Annelida | Animalia |
| 10 | G_ttingen minipig |  |  |  |  |  |  |  |
| 11 | Human | Home sapiens | Homo | Homidae | Primates | Mammalia | Chordata | Animalia |
| 12 | Lizard | Lizard | Lizard | Lacertidae | Sauria | Reptilia | Chordata | Animalia |
| 13 | Macaques | Macaques | Macaca | Cercopithecidae | Primates | Mammalia | Chordata | Animalia |
| 14 | Medaka fish | Medaka fish | Oryzias | Adrianichthyidae | Cyprinodontiformes | Actinopterygii | Chordata | Animalia |
| 15 | Rhesus monkeys | Rhesus monkeys | Macaca | Cercopithecidae | Primates | Mammalia | Chordata | Animalia |
| 16 | Mosquitoes | Mosquitoes | Anopheles/Culex/Aedes | Culicidae | Diptera | Insecta | Arthropoda | Animalia |
| 17 | Mouse | Mouse | Mus | Muridae | Rodentia | Mammalia | Chordata | Animalia |
| 18 | Naked mole-rat | Heterocephalus glaber | Heterocephalus | Bathyergidae | Rodentia | Mammalia | Chordata | Animalia |
| 19 | Nile tilapia | Nile tilapia | Nile tilapia | Cichlidae | Perciformes | Pisces | Chordata | Animalia |
| 20 | Pig | Pig breeds | Eurasian pigs | Suidae | Artiodactyla | Mammalia | Chordata | Animalia |
| 21 | Planaria | Planaria | Planaria | Planariidae | Tricladida | Turbellaria | Platyhelminthes | Protista |
| 22 | Plasmodium vivax | Plasmodium vivax | Plasmodium | Plasmodiidae | Eucoccida | Sporozoa | Apicomplexan parasites | Protista |
| 23 | Pogona vitticeps | Pogona vitticeps | Pogona | Agamidae | Souamata | Reptilia | Chordata | Animalia |
| 24 | Rat | Rat | Rattus | Muridae | Rodentia | Mammalia | Chordata | Animalia |
| 25 | Sheep | Sheep | Capra | Bovidae | Artiodactila | Mammalia | Verterbrata | Animalia |
| 26 | Xenopus laevis tadpoles | Xenopus laevis | Xenopus | Pipidae | Anura | Amphibia | Chordata | Animalia |
| 27 | Zebrafish |  |  |  |  |  |  |  |
